# Optimal treatment recommendations for diabetes patients using the Markov decision process along with the South Korean electronic health records

**DOI:** 10.1038/s41598-021-86419-4

**Published:** 2021-03-25

**Authors:** Sang-Ho Oh, Su Jin Lee, Juhwan Noh, Jeonghoon Mo

**Affiliations:** 1 Department of Information and Industrial Engineering, Yonsei University, Seoul, 03722 Republic of Korea; 2 Department of Internal Medicine, Seoul Red Cross Hospital, Seoul, 03181 Republic of Korea; 3 Department of Preventive Medicine, Yonsei University College of Medicine, Seoul, 03722 Republic of Korea

**Keywords:** Diabetes complications, Type 2 diabetes, Preventive medicine, Scientific data

## Abstract

The extensive utilization of electronic health records (EHRs) and the growth of enormous open biomedical datasets has readied the area for applications of computational and machine learning techniques to reveal fundamental patterns. This study’s goal is to develop a medical treatment recommendation system using Korean EHRs along with the Markov decision process (MDP). The sharing of EHRs by the National Health Insurance Sharing Service (NHISS) of Korea has made it possible to analyze Koreans’ medical data which include treatments, prescriptions, and medical check-up. After considering the merits and effectiveness of such data, we analyzed patients’ medical information and recommended optimal pharmaceutical prescriptions for diabetes, which is known to be the most burdensome disease for Koreans. We also proposed an MDP-based treatment recommendation system for diabetic patients to help doctors when prescribing diabetes medications. To build the model, we used the 11-year Korean NHISS database. To overcome the challenge of designing an MDP model, we carefully designed the states, actions, reward functions, and transition probability matrices, which were chosen to balance the tradeoffs between reality and the curse of dimensionality issues.

## Introduction

Medical treatment can be viewed as a series of interactions between patients and doctors. Doctors observe the state of patients through various types of examinations and choose treatments accordingly. The patients’ state changes in response to the treatment. These are called sequential decision processes since a sequence of interrelated decisions must be made over time. At a broad level, modeling of dynamic sequential decision making in medicine has a long and varied history. The Markov-based approach that we have used falls into the category of these modeling techniques, which were originally described in terms of medical decision-making by Beck and Pauker^1^. Other approaches utilize dynamic influence diagrams^2^ or decision trees^3,4^ to model temporal decisions. In all cases, the goal is to determine optimal sequences of decisions over a particular horizon.

Markov decision processes (MDPs) comprise one such efficient technique for determining optimal sequential decisions in dynamic and uncertain environments^5,6^. However, there have been relatively few applications in healthcare^5–11^. MDPs directly address many of the challenges faced in clinical decision-making^4,5^. Even though clinicians usually decide the treatment regimen taking into account patient's health condition, the effect of treatment for a given patient is unsure and this ambiguity is only exacerbated by an attempt to predict the results of a series of treatments over time.

A challenge in applying MDPs is that they require a data-intensive estimation step to generate reasonable transition and observation models. Large state/action spaces are also computationally expensive to solve, particularly in partially observable settings, and must adhere to specific Markov assumptions that the current time point (t) is dependent only on the previous time point (t − 1). Careful formulation of the problem and state space is necessary to handle such issues^4,6^.

The sharing of EHRs by the National Health Insurance Service (NHISS) of Korea has made it possible to analyze medical treatments, prescriptions, and medical check-up using Koreans’ Electronic Health Records (EHRs). After considering the merits and effectiveness of such data, we analyzed the medical information of patients and recommended the optimal pharmaceutical prescription for diabetes, known to be the most burdensome disease for Koreans. When prescribing diabetes medications, doctors would consider the patient's state such as complications, period of having diabetes, fasting plasma glucose and more. The initial medication treatment for diabetes starts from single therapy using metformin^12,13^. The doctors can move to double or triple therapy when patient's condition is not appropriate for single or double therapy respectively^14–17^. In previous studies, it is effective to choose multiple (double/triple) therapy as early as possible for maintaining proper glucose level as well as preventing complications^14,18–20^.

The goal of this study was to develop a diabetes treatment recommendation system using the EHRs of Koreans in combination with MDP which addresses the challenges stated above. The system decides to recommend whether to use single, double, or triple therapy of diabetes medication according to the state of diabetes patient. While a few previous studies have evaluated the medical recommendations for diabetes through MDP^8–11,21^, despite the challenges of MDP, their models only considered a few states and actions while proposing recommendations. To overcome these challenges, we increased the number of states and actions to as many as possible, so as to contain more information on patients and to provide various choices of recommendations for treating diabetes. In our paper, optimal diabetes medication recommendation by MDP model is the principal outcome. We recommended medication type either single, double, or triple according to the states of patient. Quality-adjusted life year (QALY) is used for reward function to quantify the quality of a year of life with the discomfort due to medical interventions such as diabetic complications. With QALY, MDP model can recommend optimal medication for each state of patient by choosing medication which has highest reward value. For diabetes patients, high blood sugar levels can serious damage many organs of our body leading to diabetic complications. Therefore, it is important to prevent diabetic complications by controlling blood sugar level well. To prevent diabetic complications, we recommended appropriate medication according to the state of patient by our MDP model. Lastly, we proved diabetic complication occurrence can be delayed by our MDP medical recommendations.

## Materials and methods

### Institutional review board statement

This study was approved by the Institutional Review Board (IRB) of Yonsei University (IRB NO. 7001988-201808-h-444-01E). This is data based research which, data was provided from National Health Insurance Sharing Service (NHISS) of Korea.

The NHISS is a national agency established to enhance the access and convenience of utilization of National Health information on data. The NHISS collects the human data in accordance with relevant guidelines and regulations which includes that informed consent was obtained from all subjects (if subjects are under 18, from a parent and/or legal guardian).

### MDP model

We used the MDP model to find the optimal prescription strategy based on patients’ health states over the course of their lifetime. Our model’s objective was to determine the optimal treatment strategy for a single patient that maximizes his/her expected discounted QALY over a planning horizon, t = 1, … , T. We adopted QALY in our model to improve the patients’ expected time in a healthy state by delaying the onset of diabetic complications.

The MDP model consists of four features: [S, A, T, R], where S is a set of states, A is a set of actions, T:S × A × S → [0,1] is a set of state transition probabilities, and R:S × A × S × R → [0,\infty] is a reward function.

### State space *S*

The state space in our model included the following five components of patients’ information:

$${S}^{t}=\left({S}_{Chronic}^{t},{S}_{Acute}^{t},{S}_{Risk}^{t},{S}_{Period}^{t},{S}_{FPG}^{t}\right) \quad \mathrm{ t}=1,\dots ,\mathrm{T}$$

${S}_{Chronic}^{t}$ is the state of having chronic complications of diabetes at time t for t = 1,…,T. If a patient has one or more chronic complication(s) at time t it is 1, or else, it is 0. We considered six chronic complications such as diabetic retinopathy, diabetic cataract, and so on, as shown in Table 1. We also stated the International Classification of Diseases (ICD-10) codes which we used to define each symptom in the dataset.

**Table 1:** Types, symptoms, and ICD-10 codes of complications.

Types of complications	Symptoms	ICD-10 codes
Chronic	Diabetic retinopathy	H36.0, E10.3, E11.3, E12.3, E13.3, E14.3
	Diabetic cataract	H28.0
	Diabetic nephropathy	N08.3, E10.2, E11.2, E12.2, E13.2, E14.2
	Chronic kidney disease	I12.0, I13.1, I13.2, N18
	Foot disease	E10.5, E11.5, E12.5, E13.5, E14.5
	Diabetic neuropathy	G59.0, G63.2, E10.4, E11.4, E12.4, E13.4, E14.4
Acute	Myocardial infarction	I21, I22
	Heart failure	I11.0, I13.0, I13.2, I50
	Hypoglycemia	E10.63, E11.63, E12.63, E13.63, E14.63

${S}_{Acute}^{t}$ is the state of having acute complications of diabetes at time t for t = 1,…,T. It is 1, if a patient has one or more of the three acute complications in Table 1, otherwise it is 0.

${S}_{Risk}^{t}$ represents whether a patient is in severe risk state of diabetes or not. It is 1 when the patient is at a severe risk state, otherwise it is 0. To assess the likelihood of a patient having diabetes, we used the Diabetes Risk Score^22^, which was designed as a screening tool for identifying high-risk individuals in the population and for increasing awareness relating to modifiable risk factors and healthy lifestyles^23^. The score is based on seven factors such as sex, age, BMI, family history, smoking, intake of hypertension medication, and intake of steroid medication. If the score is above the threshold (we have used 15 as the median value for the threshold) it is 1, otherwise it is 0. We used the diabetes risk in state space is to compress many information of patient in one state space to avoid curse of dimensionality problem in MDP and also we believed that ${S}_{Risk}^{t}$ can also reflect the severe state of patient who already has diabetes.

${S}_{Period}^{t}$ represents time elapsed since the occurrence of diabetes. If the time is less than or equal to 4 years, it is 0. If the time ranges between 5 to 8 years, it is 1, otherwise it is 2.

Lastly, ${S}_{FPG}^{t}$ represents the fasting plasma glucose (FPG) level. If the level is below 100 mg/dL, it is 0, between 100 to 125 mg/dL it is 1, and above 125 mg/dL it is 2. These three levels were divided into stages of: normal, impaired FPG level but might not have diabetes, and abnormal.

The total number of states in our model are 72 (2 × 2 × 2 × 3 × 3). Due to the state space explosion and computing power challenges, we tried to keep the model as simple as possible, whilst retaining as much information as possible.

### Action space *A*

The action of our model comprised prescriptions for diabetes patients. We selected eight possible actions as shown in Table 2, which consists of mono, dual, and triple therapies which as their names indicate, imply prescribing one, two, and three medications, respectively. Generally, monotherapy is used for less severe patients while dual and triple therapies are used for more severe patients.

**Table 2:** Action space descriptions.

Type	Action no	Description
Monotherapy	A1	Metformin (MET)
	A2	Sulfonylureas (SUL)
	A3	Others
Dual therapy	A4	Metformin + sulfonylureas (MET + SUL)
	A5	Metformin + DPP-4 inhibitors (MET + DPP4I)
	A6	Others
Triple therapy	A7	Metformin + sulfonylureas + alpha-glucose inhibitor (MET + SUL + AGI)
	A8	Others

Table 2 shows the popular medications for diabetes with corresponding frequencies. There are eight popular medications for diabetes: Metformin (MET), Sulfonylureas (SUL), Meglitinides (MEG), Thiazolidinediones (TZD), DPP-4 inhibitors (DPP4I), GLP-1 receptor agonists (GLP-1RA), and insulin. Dual therapies combine two medications out of the eight possible medications. Popular dual therapies are MET + SUL, MET + DPP4I, and so on. Popular triple therapies are MET + SUL + AGI, MET + SUL + TZD, and MET + SUL + DPP4I.

**Figure 1:**
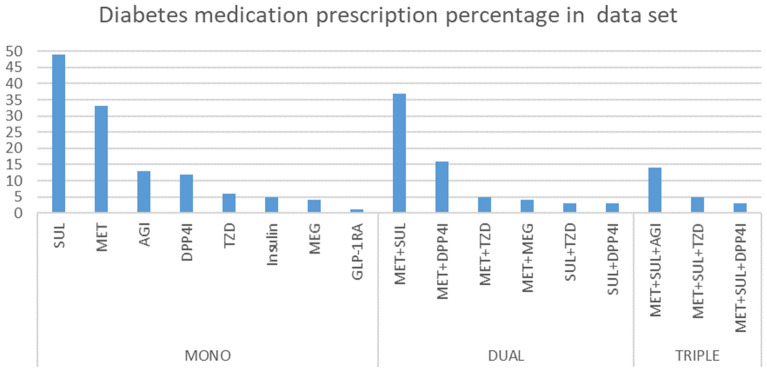
Diabetes medication prescription percentages in the dataset. This figure shows the diabetes medications prescription by frequency in our dataset. We classified the medication by number of medications prescribed (mono, dual, triple-therapy). The poll shows the percentage of each medications.

Since the number of combinations would have increased exponentially had we considered all possible prescriptions, we decided to limit the number of possible actions to overcome action space explosions. Therefore, in Fig. 1, we sorted the medications by frequency of prescription in our dataset and chose the top two medications for each type and classified other medication as “others.” However, for triple therapy we chose only one specific action (number 7) because, other than that, the other triple therapy prescription frequencies were very low to be taken as actions.

### Transition probability matrices (TPM)

In the MDP model, we needed as many transition probability matrices as the number of actions, which can be denoted by T(s,s′,a) for $s,{s}^{{\prime}}\in S, a\in A.$ To compute T(s,s′,a), we counted the number of occurrences from state *s* to state *s*′ after taking medication denoted by *a* for all $s,{s}^{{\prime}}\in S,$ and $a\in A$.

Once the counting was finished, we divided the number by the sum of the rows, so that the sum of each row becomes 1.

One issue with the TPM model was that some rows did not have any occurrences. In such cases, transition between certain states was rare. To deal with the blank state, we calculated the cosine similarity between each vector by sex, state, and actions to check if we could fill in the blank state with another matrix. Table 3 shows the results of vector similarity.

**Table 3:** Vector similarity test between gender, states, and actions.

Vector similarity test expanded in action													
	Sex	FPG	Period	Actions									
				Mono (M)			Dual (D)			Triple (T)	Combinations		
				Me-S	S-O	Me-O	Me,S-Me,D	Me,D-O	Me,S-O	Me,S,A-O	MT-DT	DT-TT	MT-TT
Male	0.9683	0.6864	0.7102	0.9545	0.9658	0.9512	0.9771	0.9564	0.9852	0.9432	0.9682	0.943	0.9712
Female				0.9379	0.9785	0.9455	0.9673	0.9667	0.9746	0.9603	0.9501	0.9697	0.9642

Me: metformin; S: sulfonylureas; D: DPP4-inhibitors; A: alpha glucose inhibitor; O: others; MT: mono therapy; DT: dual therapy; TT: triple therapy.

In Table 3, both gender and actions have similarity values above 0.9 which means they are very similar. Thus, we could obtain the value from between gender and actions to fill blank states in TPM.

### Reward *R*

The reward function $\mathrm{R}\left(\mathrm{a},{\mathrm{s}}^{{{\prime}}}\right)$ of our model is the patient’s expected discounted QALYs as shown in Eq. (1), where *a* is an action and *s*′ is its resulting state. Health services researchers typically use QALYs to quantify the quality of a year of life with the discomfort due to medical interventions. Treating or screening a patient for a chronic disease will offer some reward to the patient, such as a potentially longer life. However, these benefits come at some “cost” to the patient, whether it be a reduction in quality of life, such as side effects due to medications or discomfort due to a screening test, or a financial cost, such as medication or hospitalization expenses. The QALY is also used by many health care researchers^21,24,25^. We, too, adopted it in our model.

1 $$R\left(a,{s}^{{\prime}}\right)={R}^{WTP}\left[(1-{d}^{Chronic}\left({s}^{{\prime}}\right))(1-{d}^{Acute}\left({s}^{{\prime}}\right))(1-{d}^{Risk}\left({s}^{{\prime}}\right))(1-{d}^{Period}\left({s}^{{\prime}}\right))\right]-{C}^{MED}(a)$$

Here, ${R}^{WTP}$ is a willingness to pay for a QALY of 1 which represents a patient being in perfect health for one year without any disutilities caused by medical interventions and side effects of treatment. As the patient’s quality of life decreases due to side effects of medications or disablements from a disease, the QALY value will tend towards 0. We considered four decrement factors—${d}^{Chronic}\left({s}^{{\prime}}\right)$, ${d}^{Acute}\left({s}^{{\prime}}\right)$, ${d}^{Risk}\left({s}^{{\prime}}\right),$ and ${d}^{Period}\left({s}^{{\prime}}\right)$—due to chronic diseases, acute diseases, risk factors, and long duration in the model, details of which are shown in Table 4. Lastly ${C}^{MED}$ represents the costs of medication we used for actions^26^.

**Table 4:** Decrement values for the reward function.

Notation	Meaning	Values
${d}^{Chronic}\left({s}^{{\prime}}\right)$	Utility decrement value associated with chronic complications^27^	0.105
${d}^{Acute}\left({s}^{{\prime}}\right)$	Utility decrement value associated with acute complications^27^	0.052
${d}^{Risk}\left({s}^{{\prime}}\right)$	Utility decrement value associated with risk score^27^	1: 0.071 0: 0
${d}^{Period}\left({s}^{{\prime}}\right)$	Utility decrement value associated with the diabetes period^28^	Period (1–4): 0.081 Period (5–8): 0.095 Period (9–11): 0.108
${C}^{MED}\left(a\right)$	Cost of medications^26^	It varies depending on the actions

The values in the third column are the decrement values that we used in our study, which were taken from other studies^26–28^. For example, the patient with chronic diseases had a QALY of 0.895 (1–0.105).

### Data descriptions

We used the National Health and Insurance Service–National Sample Cohort (NHIS–NSC) data—a population-based cohort—established by the NHISS in South Korea (data accessible upon payment)^29^. The cohort provides representative, useful information regarding citizens’ utilization of health insurance and health examinations. Currently the NHISS maintains and stores national records for healthcare utilization, prescriptions, and medical check-up. Medical check-up database contains major results from medical check-up and behavior and habitual data from questionnaire. Specifically, it includes the following contents: height, weight, waist, systolic and diastolic blood pressure, fasting plasma glucose, total cholesterol, triglyceride, HDL and LDL cholesterol, history (patient him/herself and family) of stroke, heart disease, hypertension, and diabetes, smoke status, drink habit, and exercise frequency. The cohort sampled in the 2002 database was followed until 2013, only if the participants were still eligible for health insurance. For additional information, health care in South Korea is provided by a compulsory National Health Insurance (NHI). Every South Korean is eligible for this insurance until his/her death, immigrant, or working in National Intelligence Service (NIS).

### Data cleansing

**Figure 2:**
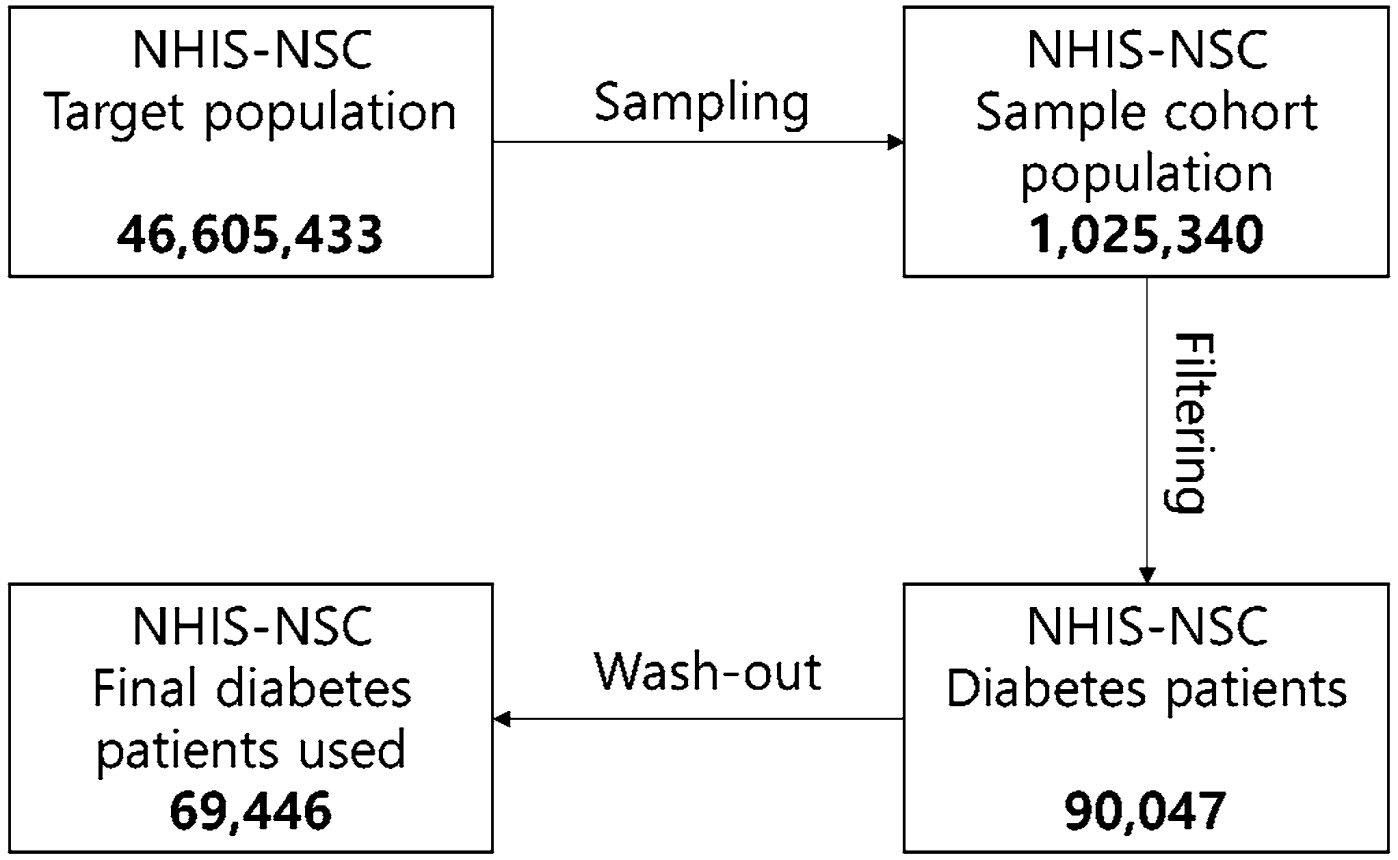
Process of data selection and number of patients included in each step. This figure shows the process of data cleansing for experiment.

Figure 2 shows the data cleaning process for maintaining proper records of diabetic patients. We selected diabetic patients’ records from the NSC data of one million that we could access from the NHISS database and filtered the patients with diabetes using the following criteria:

1. Diagnosis of diabetes according to the 10th edition of the ICD-10 codes: E10–E14.
2. Prescribed oral glucose-lowering medications for more than 30 days.

Our database finally had 90,047 patients with diabetes which met the criteria.

After filtering out diabetes patients, we also worked on the “wash-out” process. Since we did not know the exact start period of diabetes for the patients in year 2002, we deleted all their data from every other year from 2002 up to 2013, and made year 2003 as the first year of our observation. After all the processing, the total population of diabetes patients was 69,446. All the participants were followed up from January 1, 2003, until the date of their deaths or December 31, 2013, whichever occurred earlier.

### Data statistics

Table 5 shows the statistics of the 69,446 diabetes patients used for validation of the treatment recommendation system.

**Table 5:** Statistics of filtered diabetes patients in the dataset.

	Male	Female
Sex (%)	54	46
Age, mean (SD)	58.4 (25.1)	61.7 (25.4)
Period of having diabetes (years), mean (SD)	7.5 (2.7)	8.6 (2.5)
BMI (kg/m^2^), mean (SD)	25.7 (2.7)	25.4 (2.5)
FPG (mg/dL), mean (SD)	142.9 (58.1)	145.8 (58.5)
Total cholesterol (mg/dL), mean (SD)	188.5 (45.1)	192.4 (45.7)
SBP (mmHg), mean (SD)	129.2 (24.6)	134.1 (24.8)
DBP (mmHg), mean (SD)	79.6 (14.5)	81.3 (14.7)
Smoker (%)	65	48

The male and female patients comprised 54% and 46% of the data, respectively, and their mean ages were 58.4 and 61.7, respectively. Female patients’ period of having diabetes was longer than that of the male patients by 0.9 year. For both sexes, BMIs were similar, FPG levels were above the risk level as they exceeded 140 mg/dL, and total cholesterol levels were normal. However, male patients’ blood pressure was in an “elevated” stage while female patients were in the hypertension stage 1. Lastly, the male and female patients who smoked were 65% and 48%, respectively.

## Results

### Optimal treatment recommendation result

**Figure 3:**
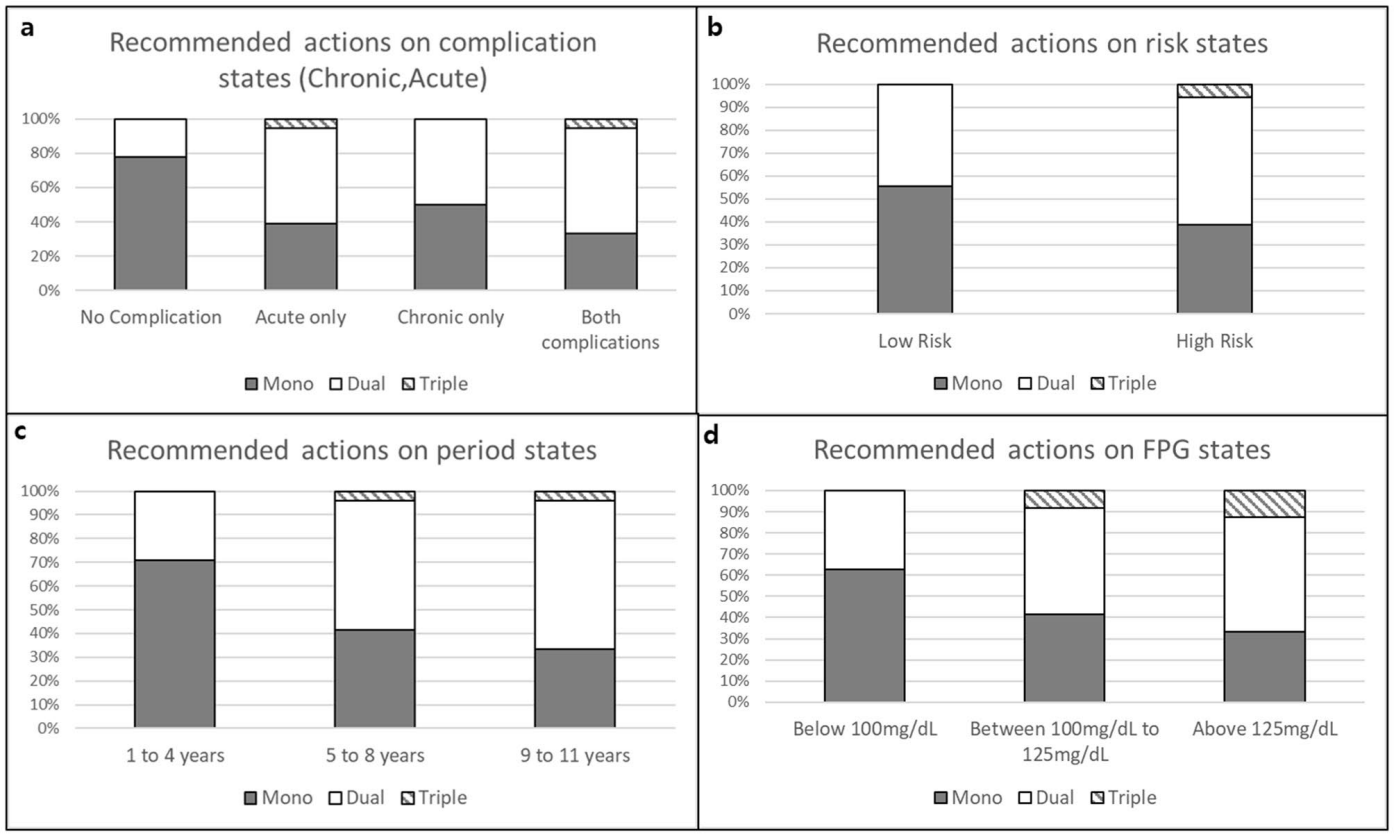
MDP recommendation actions on each state for male patients. This figure shows the recommended treatment actions according to each component of the states for male patients. (**a**) Is the recommended actions on complication states. (**b**) Is the recommended actions on risk states. (**c**) Is the recommended actions on period state and (**d**) is the recommended actions on FPG states. *Grey shaded sqaure* is for mono therapy recommendations. *Open square* is for dual therapy recommendations. And *striped square* is for triple therapy recommendations.

**Figure 4:**
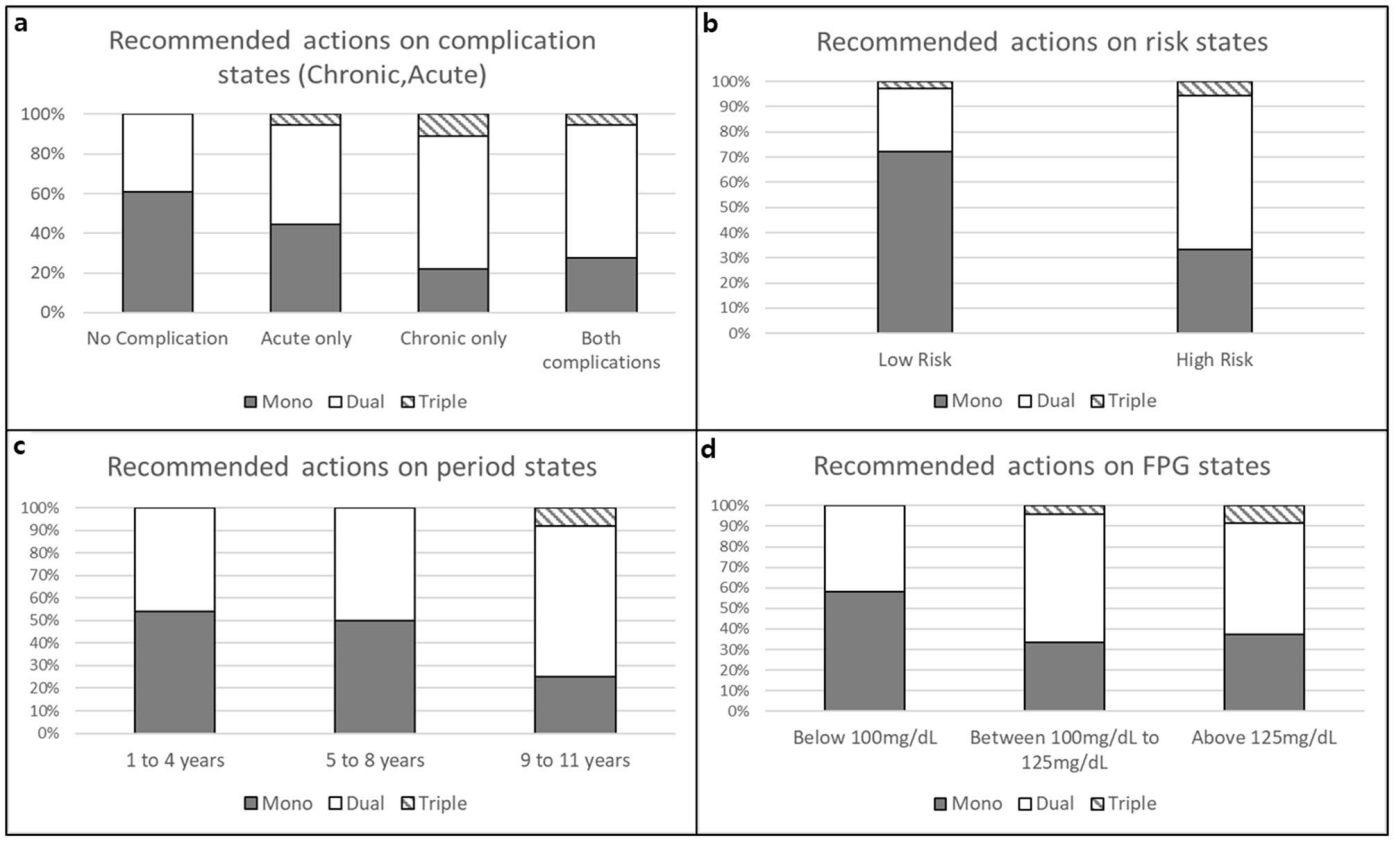
MDP recommendation actions on each state for female patients. This figure shows the recommended treatment actions according to each component of the states for female patients. (**a**) Is the recommended actions on complication states. (**b**) Is the recommended actions on risk states. (**c**) Is the recommended actions on period states and (**d**) is the recommended actions on FPG states. *Grey shaded sqaure* is for mono therapy recommendations. *Open square* is for dual therapy recommendations. And *striped square* is for triple therapy recommendations.

The recommended treatment actions according to each component of the states are shown in Figs. 3 and 4 for males and females, respectively.

Our purpose was to observe the changing trends in recommended actions as the states changed. As regards recommended actions on complication states, for the state having no complications, monotherapy and dual therapy were recommended 78% and 22% of the times, respectively, whereas when the states changed to acute, chronic, and both complications, the recommendation level increased by 30%, 28%, and 39%, respectively, for dual therapy, along with a slight increase in triple therapy. For risk states, 56% of low risk patients were recommended monotherapy, but it decreased to 39% as it progressed to high risk, and the recommendations for dual and triple therapies increased by 12% and 6%, respectively, as compared to the low risk state. In the period states, patients in the “1 to 4 years” state were recommended monotherapy and dual therapy 71% and 29% times, respectively. However, when the period state increased to “4 to 8 years” and “9 to 11 years” recommendations for monotherapy reduced to 42% and 33%, respectively, while recommendations for dual-therapy increased to 54% and 63%, respectively, and those for triple therapy increased to 4% for both states. Lastly, in the FPG state, for the normal level patients, the recommendations for monotherapy and dual therapy were 63% and 37%, respectively. For patients whose FPG levels were impaired and abnormal, the recommendations for dual-therapy increased by 12% and 16% as well as 8% and 13% for triple-therapy, as compared to patients with normal levels.

The trends of MDP recommendation results for female patients, shown in Fig. 4 above, were similar to those for male patients. In relation to complication states, monotherapy and dual therapy were recommended 61% and 39% of the times, respectively, for patients without complications, whereas for those with acute, chronic, and both complications, dual therapy was recommended 50%, 67%, and 67% of the times, respectively, and triple-therapy 6%, 11%, and 6% of the times, respectively. For risk states, 72%, 25%, and 3% of low risk patients were recommended mono, dual, and triple therapies, respectively. As it progressed to high risk, the recommendation for monotherapy decreased to 33%, while that for dual and triple therapies increased to 61% and 6%, respectively. For period states, patients in the “1 to 4 years” state were recommended monotherapy and dual-therapy 54% and 46% of the times, respectively. In the “4 to 8 years” and “9 to 11 years” states, recommendations for monotherapy reduced by 4% and 29%, respectively, while recommendations for dual therapy increased by 4% and 19%, respectively, and that for triple therapy increased to 8% for the “9 to 11 years” state. For the FPG states, patients with normal levels were recommended monotherapy and dual therapy 58% and 42% of the times, respectively, while for those whose FPG levels were impaired and abnormal, the recommendations for dual therapy increased to 63% and 54%, respectively, and for triple therapy to 4% and 8%, respectively, compared to patients whose levels were normal.

## Discussions

We validated the results of our recommendations by comparing them with doctors’ real life prescriptions. We also checked our recommendation system’s performance by observing the delay in the onset of diabetic complications. Since diabetes is usually accompanied by several complications, well-maintained diabetes could also imply the absence of complications.

### Retrospective validation

We compared the optimal recommendations of the MDP model with doctors’ real life prescriptions using the NSC data to check the similarities between the two. Since we proposed an optimal recommendation for each state, our model had 72 optimal actions.

From the NSC database, we calculated the percentage of doctor’s prescriptions for each state. The most frequent prescriptions for each state were compared with the optimal policy of the MDP model. Table 6 shows the results. Of the 72 states, 49 and 44 matched for males and females, respectively. A comparison of the MDP recommendations and doctors’ real life prescriptions revealed matching scores of 68% and 61% for males and females, respectively. The matching percentage represents the count rate which exactly matched the MDP and real life doctors’ prescriptions.

**Table 6:** Matching scores and percentages between MDP and real life prescriptions.

	Matching scores	Matching %
Males	49 out of 72	68
Females	44 out of 72	61

Based on the fact that the prescriptions may vary according to the patients’ specific conditions and doctors’ treatment styles, we believe that the actions that our MDP recommended were acceptable outcomes. To verify this, we also compared the top three real life prescriptions for each state with MDP treatment recommendations. Table 7 shows the scores when the MDP recommendations matched with the top three real life prescriptions in the dataset. Of the 72 states, the scores matched in 63 and 58 states for male and female patients, respectively, and it increased by around 20% when compared to the top three prescriptions.

**Table 7:** Matching scores and percentages between MDP and real life doctors’ prescriptions.

	Matching scores	Matching %
Male	63 out of 72	88
Female	58 out of 72	81

### A comparison of the diabetic complication occurrence periods

Patients with diabetes are at a much higher risk than others of serious complications such as coronary heart disease, stroke, blindness, kidney diseases, amputation of limbs, and neurological disorders. Thus, it is important for these patients to avoid having such complications which in addition to lessening their quality of life, could even cause death.

Accordingly, we checked if MDP recommendations could delay the occurrence of diabetic complications by comparing individuals in the cohort who followed MDP recommendations with those who did not. For research justification, we compared the group who followed MDP recommendations by matching the period of the prescription. If the real life prescription matched the MDP recommendation and its period comprised more than 50% of the patients’ whole data period, we considered them as MDP followers. Their demographics with mean and standard deviation are provided in Table 8.

**Table 8:** Demographics and risk factors for 2 groups of patients with mean and standard deviation (SD) (N = 13,973).

Gender	Male		Female	
Action	MDP followers	Other prescription takers	MDP followers	Other prescription takers
Age	53.1 (5.5)	51.9 (5.9)	57.0 (5.7)	56.4 (5.7)
Total cholesterol levels (mg/dL)	205.3 (44.8)	201.1 (43.4)	212.3 (45.6)	209.5 (43.8)
Body mass index (kg/m^2^)	24.8 (2.9)	24.9 (3.1)	25.4 (3.4)	25.5 (3.6)
Fasting plasma glucose (mg/dL)	139.8 (60.1)	142.5 (58.5)	145.2 (54.9)	143.8 (57.6)

**Figure 5:**
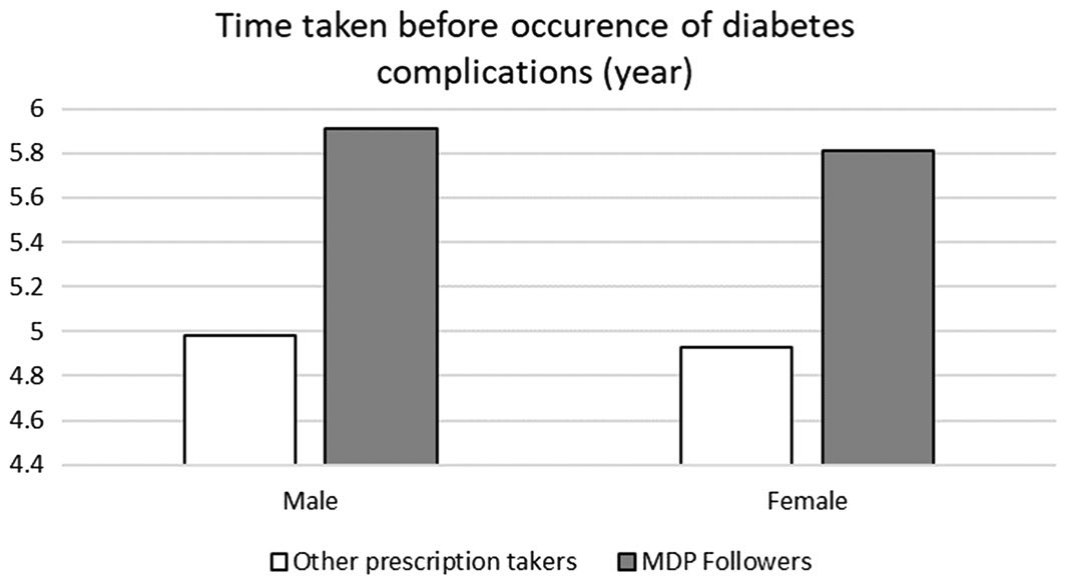
A comparison of the diabetic complication occurrence period between MDP recommendation followers and other prescription users. This figure shows the average period from the time diabetes was diagnosed to the occurrence of complications, and compared the MDP follower groups and other group. *Grey shaded sqaure* is for MDP follower group and *Open square* is for other prescription taker group.

We computed the average period from the time diabetes was diagnosed to the occurrence of complications, and compared the two groups. The results showed that male and female patients who followed the MDP recommendations appeared to have delayed the onset of diabetic complications by 0.93 year and 0.88 year, respectively, as shown in Fig. 5. This result is meaningful since the main goal of diabetes treatment is lowering glucose levels as well as avoiding complications.

The results have been verified as being statistically significant using the t-test. Since we set the alpha value at 0.05, if the p-value is less than 0.05, we can say that there is a statistically significant difference between the means of our two groups. Since both male and female patients had p = 1.46 × 10^–33^ and p = 7.59 × 10^–20^, respectively, which is < 0.05, the result is significant.

## Conclusions

We proposed an MDP-based treatment recommendation system for diabetic patients. To build the model, we used the 11-year Korean NHISS database of one million patients for each year. To overcome the challenge of designing an MDP model, we carefully designed the states, actions, reward functions, and transition probability matrices, that were chosen to balance the trade-offs between reality and the curse of dimensionality issues.

Our results show that diabetes medication recommended by our MDP system is realistic as it correctly recommends the changing trends from monotherapy to dual and triple therapies, as the patients’ state deteriorates from normal to serious. Doctors, too, increase the number of medications and prescribe them in combination when the patients’ state does not improve. To check the validity of our results, we compared the MDP-based recommendation results with real life prescriptions. Considering that prescription could vary according to the specific condition of patients and treatment styles of doctors, we compared the top three real life prescriptions in each state with MDP treatment recommendations, and obtained 88% and 81% matches for male and female patients, respectively. Lastly, we proved that our MDP recommendation can maintain better health condition by delaying the occurrence of diabetic complications. The patients who followed MDP recommendations were able to delay the onset of complications longer than those who did not follow MDP recommendations. We believe that our proposed MDP recommendation system could help doctors to prescribe appropriate diabetes medications.

## Data Availability

The datasets generated during and/or analyzed during the current study are not publicly available due to containing potentially identifying patient information collected by National Health Insurance Sharing Service which requires payment for access. But are available from the corresponding author on reasonable request.
